# Hypofractionated radiotherapy as a salvage treatment for recurrent hepatocellular carcinoma with inferior vena cava/right atrium tumor thrombus: a multi-center analysis

**DOI:** 10.1186/s12885-019-5870-3

**Published:** 2019-07-05

**Authors:** Jinrong Lou, Yong Li, Kangning Liang, Yutian Guo, Changlong Song, Lei Chen, Lifang Wang, Fei Wang, Li Zhang, Xia Chen, Xiangdong Xu, Mianshun Pan

**Affiliations:** 1 Department of Cardiology, Jiading District Central Hospital Affiliated Shanghai University of Medicine and Health Sciences, 1 Chengbei Road, Shanghai, 201800 China; 2 Center of Radiation Oncology, Wujing Hospital, 380 Hongxu Road, Shanghai, 201103 China; 3 Center of Radiation Oncology, Guangdong Nongken Central Hospital, 2 Renmin Road, Guangzhou, 524002 China

**Keywords:** Hepatocellular carcinoma, Inferior vena cava, Right atrium, Tumor thrombus, Hypofractionated radiotherapy

## Abstract

**Background:**

Recurrent hepatocellular carcinoma (HCC) with a tumor thrombus (TT) extending into the inferior vena cava (IVC)/right atrium (RA) is generally regarded as a terminal-stage condition and there is no worldwide consensus on the proper management of this situation. In the present study, we report the efficacy of hypofractionated radiotherapy (HFRT) as a salvage treatment for recurrent HCC with IVC/RA TT.

**Methods:**

We retrospectively reviewed 75 HCC patients with an IVC/RA TT who were referred for HFRT at three institutions between 2008 and 2016. 57 cases had a TT located in the IVC (IVC group), and 18 cases had a TT located in the IVC and RA (IVC + RA group). HFRT was designed to focus on the TT with or without the primary intrahepatic tumors.

**Results:**

In all cases, the TT completely disappeared (CR) in 17 patients (22.7%), 55 patients (73.3%) had a partial response (PR), and 3 patients (4.0%) had a stable disease (SD). There were no cases of progressive disease (PD). The 1-, 2-, and 3-year overall survival rates of the 75 patients were 38.7% (29/75), 13.3% (10/75) and 5.3% (4/75), respectively. The overall median survival time was 10 months. The mean survival times for the IVC group and IVC+ RA group were 13.8 ± 1.1 and 11.6 ± 2.5 months, respectively. There was no significant difference in survival between the two groups (*p* = 0.205). Log-rank test revealed that factors predicting poor survival were Child-Pugh B liver function classification, AFP ≥ 400 μg/L, intrahepatic multiple tumors, distant metastases, only the TT as the target, a biological effective dose (BED) < 55 Gy and no chance of further radiotherapy.

**Conclusions:**

HFRT appears to be an effective and reasonable treatment option for recurrent HCC patients with IVC/RA TT. The location of the tumor thrombus, either in IVC or in IVC and RA, is not the factor that influences the efficacy of radiotherapy or survival.

## Background

Large vascular invasion is a common feature of recurrent hepatocellular carcinoma (HCC). The presence of portal vein (PV) or hepatic vein tumor thrombus (TT) is frequent in these patients [1]. TT can progress along the venous wall to the inferior vena cava (IVC) or even to the right atrium (RA) [1, 2]. IVC/RA TT is found in 3–4% of HCC patients, but it seems to be increasingly discovered as a result of longer survival of HCC patients and advances in imaging techniques [3–5]. This situation may result in distant metastasis, pulmonary embolism or cardiac outflow tract obstruction, so the prognosis is usually extremely poor, and the median survival is only 1.9 months [6], which is worse than that of patients with a PV TT [7–9]. As such, the IVC/RA TT should be removed as soon as possible [10].

Although surgical removal of a TT may be effective, it is rarely performed because of the limited normal hepatic volume and higher surgery risk in these recurrent HCC patients [2–9]. Some selected patients can choose transarterial chemoembolization (TACE), but they always got unsatisfactory effect and severe complications [3, 11, 12]. In the present study, we report the efficacy of hypofractionated radiotherapy (HFRT) as a salvage treatment for recurrent hepatocellular carcinoma with IVC/RA TT.

## Methods

### Patients and diagnoses

Between January 2008 and May 2016, 1897 patients underwent HFRT for HCC at the Center of Radiation Oncology of Shanghai Wujing Hospital, Guangdong Nongken Tumor Hospital and Shanghai Jiading Central Hospital. Of these, 104 patients (5.5%) were diagnosed with advanced HCC with IVC/RA TT. There were 78 cases (4.1%) with TT located in the IVC and 26 cases (1.4%) with TT located in both the IVC and RA. Among them, 75 cases were recurrent HCC patients who received at least one definitive treatment for intrahepatic primary tumors and had complete follow-up imaging and data. Prior treatments included surgical resection, TACE, radiofrequency, radiotherapy and sorafenib. HCC was diagnosed by histopathological examination or typical manifestations in imaging [13].

### Hypofractionated radiotherapy

Hypofractionated radiotherapy was delivered by a stereotactic gamma-ray therapeutic system (SGS-I, Huiheng Medical, Shenzhen, China). A stereotactic frame and 18 Co^60^ sources in this system can deliver a high-precision radiotherapy on the target volume [14]. Each patient was immobilized by a vacuum bag and underwent 2 phases of enhanced CT simulation under a normal breathing situation with a 5 mm CT-slide thickness. This 2-phase scan had an initial interval of 2.5 mm. The 2-phase images were fused into the planning system with the CT-slide thickness of 2.5 mm, accounting for tumor motion. The gross tumor volume (GTV) was defined by the hyperdense area of the intrahepatic primary tumor during the arterial phase and/or the hypodense filling defect area of the tumor thrombus during the venous phase. The clinical target volume (CTV) expanded the GTVs of the primary tumors and tumor thrombus by a margin of 5 mm and 0 mm, respectively. A margin of 5 mm to the CTV was inclued in the planning target volume (PTV). Taking into account the size of the tumor and the patient’s liver function, radiotherapy was designed to target only the tumor thrombus in 29 patients and both the tumor thrombus and primary tumors in 46 patients. A dose of 3–4 Gy was delivered once a day and five times per week, with a total dose of 30–48 Gy (the median dose is 38Gy). The biological effective dose (BED) was evaluated by the equation: BED = nd [1 + d/(α/β)]. The mean 50% of the non-cancerous liver volume received a dose of <25Gy, The V_30_ of the uninvolved heart was < 20%. During radiotherapy, the patients underwent routine physical examinations and blood tests every week.

### Response evaluation and follow-up

**Fig. 1:**
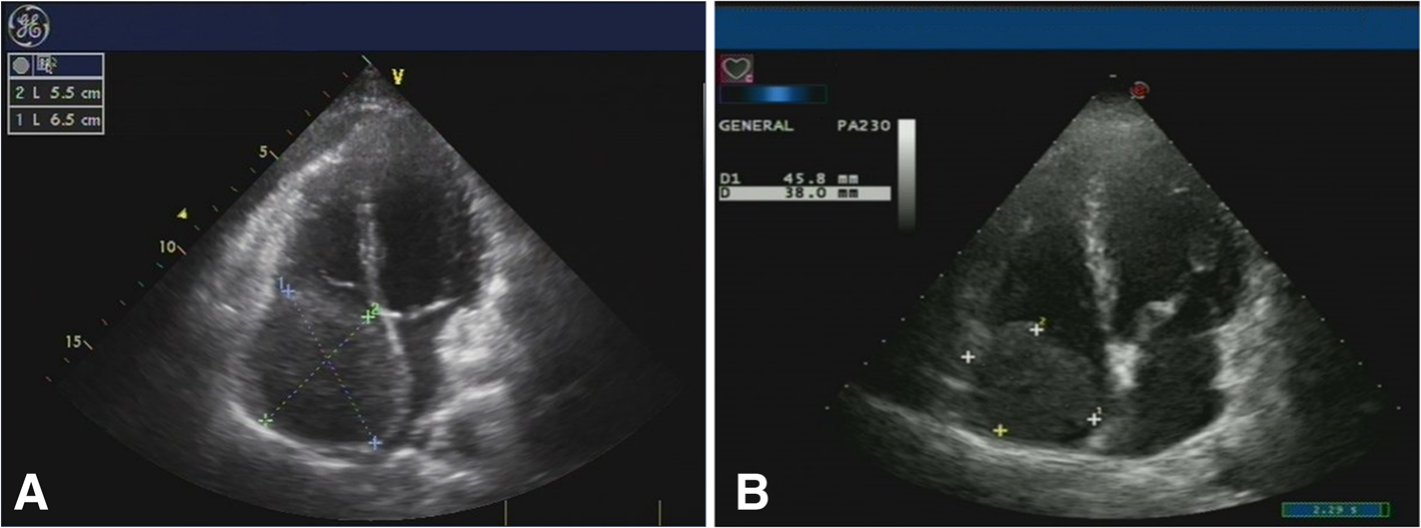
Doppler ultrasound follow-up images of one RA TT case. **a** Before HFRT, the TT (5.5*6.5 cm) filled the right atrium. **b** 14 days after completion of RT, the TT (3.8*4.6 cm) was decreased in size

**Fig. 2:**
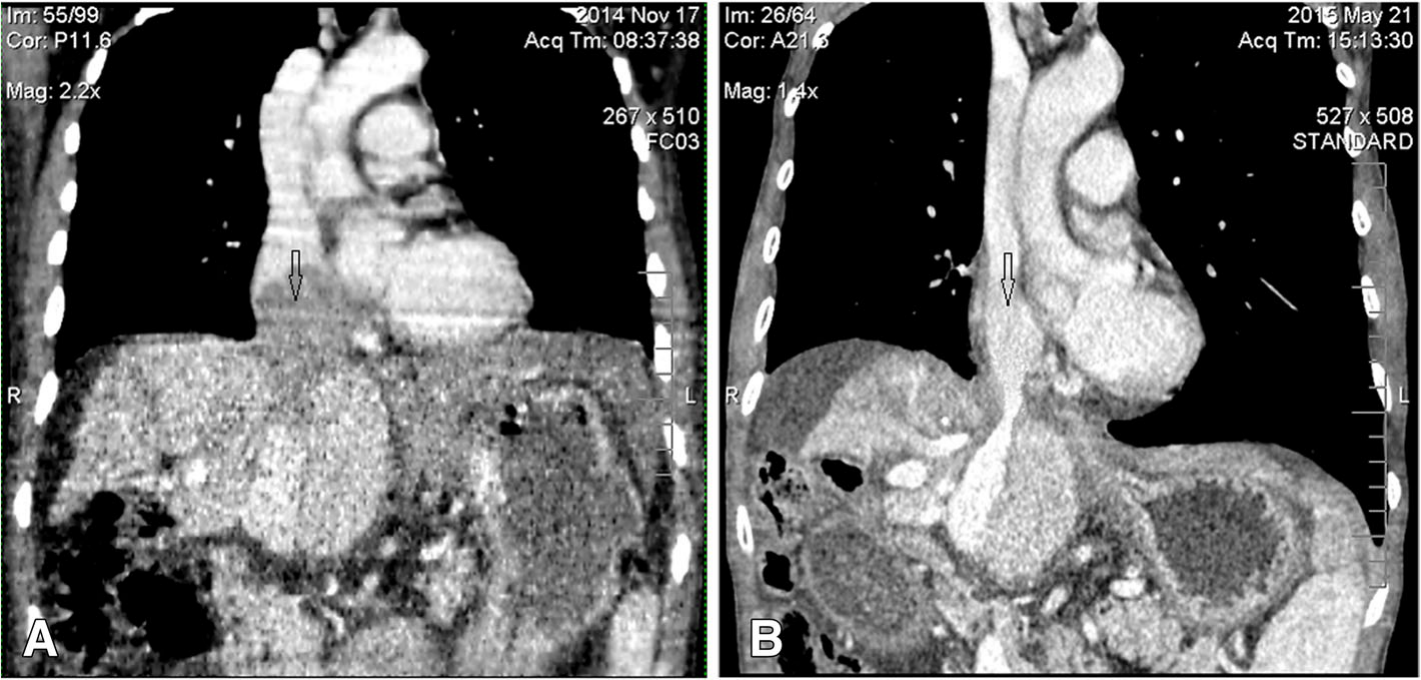
Computed tomography (CT) follow-up images of one RA TT case. **a** Before HFRT, the TT (7.5*6.5 cm) filled the right atrium, resulting in circulatory disorders and cardiac enlargement. **b** 6 months after completion of RT, the TT disappeared and circulation returned to normal

Due to the short survival of these patients and the risk of sudden death by tumor thromboembolism, the first follow-up visit was chosen to occur 0.5–1.0 months after completion of radiotherapy. At that visit, a response evaluation to the therapy was confirmed by Doppler ultrasound (Fig. 1). The CT/MRI scans were performed every 2–3 months during the first year and every 6 months thereafter (Fig. 2). The follow-up period ranged from 3 to 40 months (median, 12 months). Follow-up evaluations consisted of physical examinations, blood tests for tumor markers, liver function tests and complete blood cell count. Myocardial enzymes were detected in patients with RA tumor thrombus.

Based on CT/MRI imaging, local tumor control was classified as complete response (CR) that is complete disappearance of all measurable lesions; partial response (PR) that is 50% reduction in the sum of the perpendicular diameters of all measurable lesions; progressive disease (PD) that is 25% increase in the sum of the perpendicular diameters of all measurable lesions or the development of new lesions; Patients whose disease did not meet the criteria for either a PR or PD were classified as having stable disease (SD). Objective response rate (ORR) was defined to include those patients with CR and PR.

### Statistical analysis

The normality of each continuous variable was verified by the Kolmogorov Smirnov test and the variables were compared by an independent sample *t*-test. Categorical variables were compared either by chi-squared test or Fisher’s Exact Test. Kaplan-Meier method was used for the survival analysis and statistical significance determined by the log-rank test. Statistical analysis was performed with SPSS 19.0 software (SPSS, Chicago, IL).

## Results

### Population characteristics

The patients’ characteristics are showed in Table 1. The ratio of men and women is.

**Table 1:** The clinical characteristics of the two groups

Variables	IVC (*n* = 57)	IVC + RA (*n* = 18)	*p*
Age (years)	54.6 ± 10.6	51.9 ± 10.3	0.340
Gender			
Male	50 (87.7%)	16 (88.9%)	0.631
Female	7 (12.3%)	2 (11.1%)	
Performance status			
0	14 (24.6%)	3 (16.7%)	0.543
1	43 (75.4%)	15 (83.3%)	
Child-Pugh classification			
A	51 (89.5%)	15 (83.3%)	0.678
B	6 (10.5%)	3 (16.7%)	
Intrahepatic tumor number			
Solitary	43 (75.4%)	11 (61.1%)	0.238
Multiple	14 (24.6%)	7 (38.9%)	
LN metastases			
Absent	53 (93.0%)	15 (83.3%)	0.348
Present	4 (7.0%)	3 (16.7%)	
Distant metastases			
Absent	44 (77.2%)	10 (55.6%)	0.075
Present	13 (22.8%)	8 (44.4%)	
HBsAg			
Negative	5 (8.8%)	1 (5.6%)	0.555
Positive	52 (91.2%)	17 (94.4%)	
AFP (μg/L)			
< 400	31 (54.4%)	13 (72.2%)	0.180
≥ 400	26 (45.6%)	5 (27.8%)	
No. of prior therapies	3.7 ± 1.5	4.1 ± 1.7	0.407

*Abbreviations*: *IVC* inferior vena cava, *RA* right atrium, *LN* lymphonodus, *AFP* α-fetoprotein

66: 9 and the median age is 53.0 years (range, 23–75 years). 57 cases had a tumor thrombus located in the IVC (IVC group), and 18 cases had a tumor thrombus located in the IVC and RA (IVC + RA group). In total, 92.0% patients had underlying chronic liver disease induced by hepatitis B virus infection. Most patients (77.3%) had obvious symptoms including edema of lower extremity, abdominal pain and distention. The performance status (PS score) of the IVC group showed a slight advantage, but there was no significant difference between the two groups (*p* = 0.543). Because of major vascular invasion, all patients belonged to BCLC (Barcelona Clinic Liver Cancer staging) Stage C. In all cases, the tumor thrombus was contiguous with the intrahepatic primary tumors. The lymph node metastasis rate was 7.3% (7/75), and the distant metastasis rate was 28.0% (21/75). The numbers of prior therapy for the intrahepatic primary tumors were 3.7 ± 1.5 and 4.1 ± 1.7 for IVC and IVC + RA group, respectively. There was no significant difference in the baseline characteristics between the two groups.

### Response to treatment

At the end of the study, out of all the 75 patients who received RT, the TT completely disappeared (CR) in 17 patients (22.7%). In addition, 55 patients (73.3%) had a PR, and 3 patients (4.0%) had SD. No PD was found. The ORR was 96%. Comparing the response rate for IVC vs. IVC + RA TT, the rate of CR, PR and SD were 26.3% (15/57) vs. 11.1% (2/18), 70.2% (40/57) vs. 83.3% (15/18), and 3.5% (2/57) vs. 5.6% (1/18), respectively. There was no significant difference between the two groups (*p* = 0.370). During the follow-up, all of the patients developed intrahepatic progression, 76.1% (35/46) were within the radiation field and 23.9% (11/46) were other intrahepatic metastases. 37 cases had received further HFRT treatment (Table 2).

**Table 2:** Log-rank test for characteristics of survival in 75 patients

Variables	*n*	OS (mo)	log-rank test *P* values
Gender			
Female	9	12.2 ± 1.79	0.753
Male	66	13.4 ± 1.15	
Age			
< 50	27	12.5 ± 1.43	0.613
≥ 50	48	13.6 ± 1.38	
Child-Pugh classification			
A	66	13.9 ± 1.09	< 0.001
B	9	6.4 ± 0.80	
Performance status			
0	17	14.3 ± 2.39	0.728
1	58	12.9 ± 1.11	
HBsAg			
Negative	6	15.5 ± 2.41	0.462
Positive	69	12.9 ± 1.09	
AFP (μg/L)			
< 400	44	14.8 ± 1.59	0.041
≥ 400	31	10.9 ± 0.92	
No. of prior therapies			
< 4	35	12.8 ± 1.43	0.648
≥ 4	40	13.6 ± 1.44	
Intrahepatic tumor number			
Solitary	54	15.1 ± 1.25	< 0.001
Multiple	21	7.6 ± 0.59	
LN metastases			
Absent	66	13.6 ± 1.11	0.223
Present	9	10.6 ± 2.51	
Distant metastases			
Absent	55	14.7 ± 1.29	0.001
Present	20	8.8 ± 0.58	
PTV			
TT	29	7.6 ± 0.61	< 0.001
TT + PT	46	16.3 ± 1.34	
BED Dose (Gy)			
< 55	43	10.5 ± 0.97	0.001
≥ 55	32	16.6 ± 1.76	
Further radiotherapy			
Yes	37	17.3 ± 1.58	< 0.001
No	38	8.6 ± 0.59	
Thrombus location			
IVC only	57	13.8 ± 1.13	0.205
IVC + RA	18	11.6 ± 2.48	

*Abbreviations*: *OS* overall survival, *AFP* α-fetoprotein, *LN* lymphonodus, *TT* tumor thrombus, *PTV* planning target volume, *PT* primary tumors, *BED* biological effective dose, *IVC* inferior vena cava, *RA* right atrium

### Survival

**Fig. 3:**
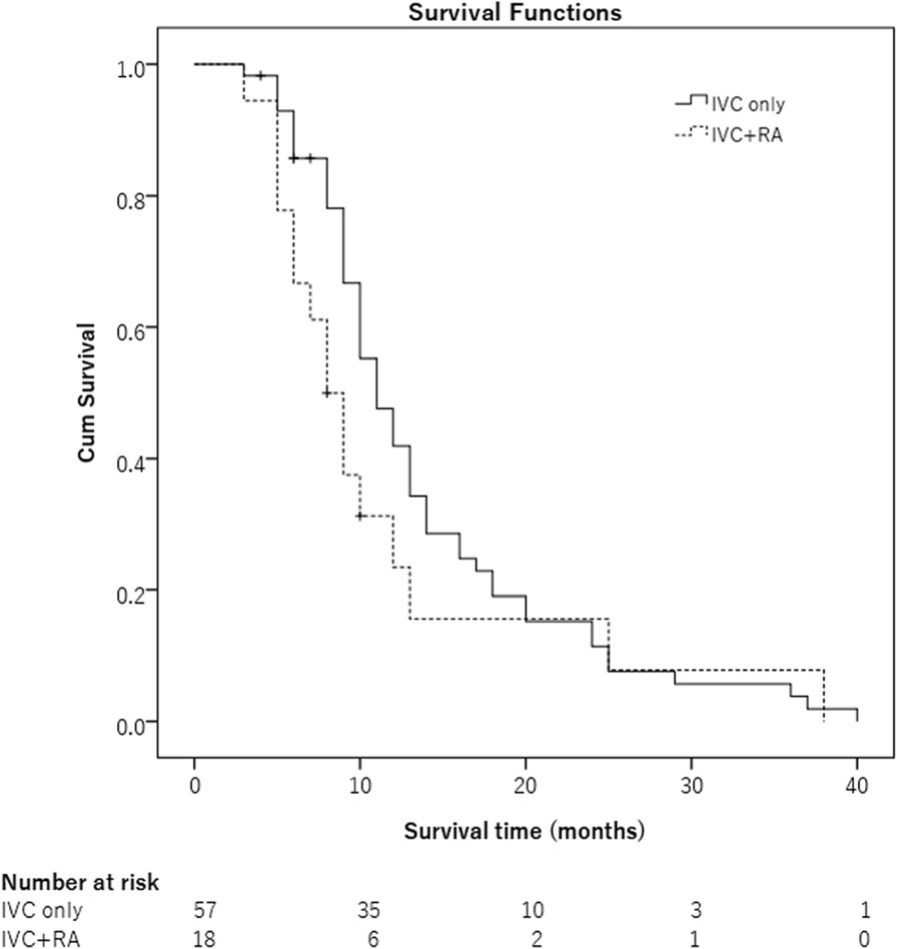
IVC TT group and IVC + RA TT group did not have a significant difference in survival (*P* = 0.205)

At the end of this study, 69 patients (92%) had died, and 6 patients (8%) were alive: 4 patients in the IVC TT group and 2 patients in the IVC + RA TT group. The causes of death included intrahepatic tumor progression in 60 (87.0%) patients, esophageal varices bleeding in 5 (7.2%) patients, brain metastases in 3 (4.3%) patients, and brainstem hemorrhage in 1 (1.4%) patient. The 1-, 2-, and 3-year overall survival rates of the 75 patients were 38.7% (29/75), 13.3% (10/75) and 5.3% (4/75), respectively; the overall median survival time was 10 months. The survival for patients in IVC TT group was 43.9% (25/57) at 1 year, 14.0% (8/57) at 2 years, and 5.3% (3/57) at 3 years, with a median survival time of 11 months; the longest survival was 40 months. The survival of patients in the IVC + RA TT group was 22.2% (4/18) at 1 year, 11.1% (2/18) at 2 years, and 5.6% (1/18) at 3 years, with a median survival time of 8 months. The longest survival time was 38 months. The mean survival time for the IVC TT only group was 13.8 ± 1.1 months, and for the IVC + RA TT group was 11.6 ± 2.5 months. As shown in Fig. 3, the two groups did not have a significant difference in survival (*p* = 0.205).

Log-rank test revealed that higher α-fetoprotein (AFP) level, Child-Pugh B liver function classification, multiple intrahepatic tumors, distant metastasis, only the TT in the PTV, BED < 55 Gy and no further radiotherapy were significantly associated with a worse prognosis. We noted that for the patients receiving RT, the location of the tumor thrombus, either in IVC or in IVC + RA, did not influence the survival. If the PTV included the TT and primary tumor, the BED dose was at least 55 Gy or the patients had the chance to receive further radiotherapy, the mean survival time was more than 16 months (Table 2).

### Toxicity

22 patients (29.3%) suffered from transient acute upper gastrointestinal toxicities. According to the grading system of the Radiation Therapy Oncology Group, 18 patients were assessed as grade 1 and 4 patients were assessed as grade 2. For the patients with a RA tumor thrombus, there was no significant increase in the myocardial enzymes after radiotherapy. Radiation-induced liver disease, pulmonary embolism or sudden cardiac arrest was not observed.

## Discussion

Liver cancer is a commonly diagnosed cancer and second leading cause of cancer death worldwide in men and in less developed countries, with China alone accounting for approximate 50% of the total number of cases and deaths [15, 16].

HCC is the most common (70 to 85%) histological type of primary liver cancer [17]. The treatment for this disease usually requires a multiple management strategy with different rewards and risks from each method [18]. Unfortunately, most patients are still suffering from tumor recurrence. When recurrent HCC does occur, the tumor tends to be more aggressive and harder to treat, and a hepatic/portal vein TT is very common [19]. However, a TT extending into the IVC/RA is reported only in 0.67 to 4.8% HCC patients [20–22]. In this study, the rates of IVC TT and IVC + RA TT were 5.5 and 1.4%, similar to previous report. With further prolongation of survival time in HCC patients, the incidence of a TT is increasing.

HCC with an IVC/RA TT is generally regarded as a terminal-stage condition and there is no worldwide consensus on the proper management of this situation [23]. The 1-year survival rates of some palliative treatments such as chemotherapy, TACE and radiofrequency ablation range from 7 to 18% [24–26]. Although surgery has long been attempted for this situation, its therapeutic effect seems unsatisfactory [27]. Clinically, to guide surgical options, IVC and RA TT are usually classified into three types: type I when the IVC TT is below the diaphragm; type II when the IVC TT is above the diaphragm and under the atrium; and type III when the TT has entered into the right atrium. For type III, the operation should be performed by liver and cardiothoracic surgeons [28]. However, although underwent curative resection, the median recurrence-free survival of these patients was only 3.8 months [29]. Nevertheless, the initial operation can reduce the tumor burden quickly and offer these patients the chance for subsequent multidisciplinary treatments, such as TACE, radiotherapy, local ablation and so on, which may help reduce tumor recurrence and improve survival time [27].

Radiotherapy is a very important treatment for liver cancer. Modern radiation techniques have greatly reduced the risk of radiation-associated disease by delivering a high dose to the target, with a sharp dose gradient to the adjacent normal tissues [30, 31]. Clinical outcomes of radiotherapy for HCC TT are encouraging and patients with an isolated IVC TT may have significant higher response rates and longer survival compared to those with a PV TT [12]. Radiotherapy may be of a strongly protective factor and help to prolong survival of HCC patients with TT [32].

Because of the low incidence, the clinical outcomes of RT for IVC and RA tumor thrombus are rarely reported. Duan [33] ever reported that a combination of radiotherapy and TACE was more effective in the control of HCC IVC/RA TT and no obviously severe complications. In our study, all the cases were recurrent HCC. Overall, 94.6% (71/75) of cases received TACE. Unfortunately, in each case, tumor thrombus was still in progress. After salvage radiotherapy, the ORRs were 96.5% (55/57) and 94.4% (17/18) for IVC and IVC + RA tumor thrombosis, respectively. No PD cases were found. The median survival times were 11 and 8 months for patients in the IVC and IVC + RA tumor thrombosis groups, respectively. There was no significant difference in survival (*p* = 0.205). The location of the tumor thrombus, for either in IVC or in IVC + RA, did not influence survival. Log-rank test revealed that AFP ≥ 400 μg/L, Child-Pugh B liver function, multiple intrahepatic tumors, distant metastasis, only the TT in the PTV, BED < 55 Gy and no further radiotherapy were found to be significantly associated with a worse prognosis. However, when the PTV included the TT and primary tumor, the BED dose was at least 55 Gy or the patients had the chance to receive further radiotherapy, the mean survival time was more than 16 months.

Although there is a risk of thrombus dislodgment, we never encountered this during radiotherapy. One reason may be that the tumor thrombus was covered by endothelium and subsequently adherent to the wall of the IVC or the endocardium [20]. Another reason may be that the tumor thrombus was a contiguous extension of the intrahepatic HCC. Finally, the TT is frequently supplied by the hepatic artery and some extrahepatic arteries [3–33], these blood vessels may serve as a “tree root” and stabilize the TT.

The limitations of this study include its small number of patients and the absence of liver function index of retention rate of indocyanine green 15 min after administration (ICGR15) because of its retrospective design. In addition, the late toxicity was difficult to assess because of the short survival time of these patients. Although with these deficiencies, HFRT appears to be a reasonable treatment for recurrent HCC patients with IVC/RA tumor thrombus due to its high efficacy and low toxicity. However, the question of why IVC/RA TT is a radiation sensitive lesion still needs further study.

## Conclusions

In summary, HFRT appears to be an effective and reasonable treatment option for recurrent HCC patients with IVC/RA TT. Patients with Child-Pugh classification A, a PTV including the TT and primary tumor, a delivered BED dose ≥55 Gy and the opportunity to receive further radiotherapy may obtain a survival benefit. The location of the tumor thrombus was not a factor that influenced the efficacy of radiotherapy or survival.

## Data Availability

Additional data and materials may be requested from the corresponding author on reasonable request.

## Abbreviations

3D-CRT: Three-dimensional conformal radiotherapy
AFP: α-fetoprotein
BED: Biological effective dose
CPB: Cardiopulmonary bypass
CR: Completely disappeared
CT: Computed tomography
CTV: Clinical target volume
GTV: Gross tumor volume
HCC: Hepatocellular carcinoma
HFRT: Hypofractionated radiotherapy
ICGR15: Retention rate of indocyanine green 15 min after administration
IMRT: Intensity modulated radiotherapy
IVC: Inferior vena cava
MRI: Magnetic resonance imaging
OS: Overall survival
PD: Progressive disease
PET: Positron emission tomography
PR: Partial response
PTV: Planning target volume
PV: Portal vein
RA: Right atrium
SD: Stable disease
TACE: Transarterial chemoembolization
TT: Tumor thrombus
